# 基于蛋白质组学的肺癌生物标志物研究进展

**DOI:** 10.3779/j.issn.1009-3419.2015.06.11

**Published:** 2015-06-20

**Authors:** 辉 谢, 振岗 陈, 广舜 王

**Affiliations:** 301800 天津，天津医科大学宝坻临床学院肿瘤外科 Baodi Clinical Hospital, Tianjin Medical University, Tianjin 301800, China

**Keywords:** 肺肿瘤, 蛋白质组学, 生物标志物, Lung neoplasma, Proteomics, Biomarker

## Abstract

蛋白组学技术可以应用于癌症研究来检测差异蛋白质表达以发现癌症生物标志物。肺癌的生物标志物在肺癌早期诊断、指导治疗和预后监测方面起着关键作用。因此，迫切需要确定新的早期诊断和预后指标以开辟新的治疗途径。本文简要介绍了基于蛋白质组学的肺癌生物标志物的最新研究报告。他包括作为诊断、预后和预测性的生物标志物，以及基于最近发表文献的基础上和我们所做的相关工作的总结。

## Abstract (en)

Proteomic technologies can be applied to cancer research to detect differential protein expression that could find cancer biomakers. Lung cancer biomarker discovery is significant due to its anticipated critical role in early diagnosis, therapy guidance, and prognosis monitoring of lung cancer. Therefore, there is an indeed need to identify new biomarkers for early diagnosis and prognosis that could serve to open novel therapeutic means. This article briefly introduces the latest reports in proteomic studies of lung cancer. It contains diagnostic, prognostic, and predictive biomarkers, and a summary based on the most recent literature and our own work.

蛋白质组学（Proteomics）作为一门方法学，用于鉴定出某一研究对象的全部蛋白。其目的是从整体的角度分析其蛋白质组成成分、表达水平与修饰状态，了解蛋白质之间的相互作用与联系，揭示蛋白质功能与细胞生命活动的规律，已经成为研究肿瘤生物学不可或缺的工具。任何一种疾病在表现出可察觉的症状之前，就已经有一些蛋白质发生了变化，因此对各种疾病的关键蛋白和标记蛋白的深入研究，为最终找到疾病的病因、发病机制提供了客观依据，也是疾病临床分期分型的分子基础。因此寻找敏感性高、特异性好的肺癌生物标志物已成为现阶段需要解决的重要问题。

## 1 简介

肺癌在20世纪初是一种少见的疾病，但随着环境和生活方式的改变，目前肺癌已成为发病率和死亡率最高的癌症之一。大约75%的肺癌患者被确诊时已属晚期，伴随局部和/或远处转移，错过了最佳治疗时机，5年生存率不足15%，已成为全球范围内恶性肿瘤死亡的最常见病因^[1]^。同时使用组织学分类有他的不足之处，因为他没有考虑到肺肿瘤的异质性^[2]^。例如，小细胞肺癌（small cell lung cancer, SCLC）和非小细胞肺癌（non-small cell lung cancer, NSCLC）之间或在NSCLC亚型之间重要的分子和遗传学改变不同，意味着对肺癌患者进行的治疗方法也不同^[3]^。例如*PI3KCA*基因扩增更多出现在肺鳞癌，*KRAS*和*LKB1*基因突变在肺腺癌中更为常见^[4]^。因此有必要挖掘出有助于肺癌早期诊断和治疗的新的生物标志物和治疗靶点。作为细胞的结构基础和实际的功能执行者，蛋白质可以提供一些DNA和RNA不能提供的信息。因此，包括蛋白质的分离、质谱鉴定及生物信息学分析等技术的蛋白质组学方法将打开一个新的窗口，使我们更好地理解这种疾病。目前蛋白质组学技术已实现在一个微小肿瘤组织块中，找到癌、癌旁组织的大部分蛋白，其覆盖度达到了RNA-seq对转录组检测的水平；并能实现对磷酸化蛋白的检测，通过对差异蛋白及磷酸化位点的分析，可以在单个肿瘤中发现起关键驱动作用的蛋白及其信号通路。所以在癌症生物标志物和治疗靶点的鉴定方面，蛋白质组学正成为一个越来越重要的工具。在本文中，将描述集中在使用如二维凝胶电泳、液相色谱和质谱法等不同的蛋白质组技术鉴定肺癌患者组织、体液等的差异表达蛋白所得到的诊断、预后和预测相关性的肺癌标志物，这可能会有助于改善对这种疾病的认识。

## 2 诊断性生物标志物

诊断性生物标志物必须直接与疾病的存在相关，并且在疾病的早期阶段特异性和敏感性较高^[5]^。目前，在高危人群中，通过螺旋计算机断层扫描（computed tomography, CT）在肿瘤检测中是一种很有用的工具。通过组织、血、尿等检测到的蛋白质生物标志物，可能是对CT早期检测的一种补充。

### 2.1 NSCLC患者的组织样品中检测到的诊断性生物标志物

人类癌症的组织特异性生物标志物的发现对早期诊断和疾病的分子认识至关重要。通过蛋白质组学技术鉴定癌组织和正常肺组织之间蛋白水平表达的差异，检测诊断肺癌的生物标记物。目前为止，已鉴定了很多蛋白。亲环素A（cyclophilin, CYP-A）、巨噬细胞移动抑制因子（macrophage migration inhibitory factor, MIF）^[6]^、聚免疫球蛋白受体（polymeric immunoglobulin receptor, PIGR）、14-3-3η^[7]^、胸腺素β4（thymosin β4, TMSB4）、泛素、酰基辅酶A结合蛋白（acyl-CoA-binding protein, ACBP）、半胱氨酸蛋白酶抑制剂A（cysteine protease inhibitor A, CSTA）、细胞色素C^[8]^等已经被确定为肺癌诊断性生物标志物。MIF和14-3-3η蛋白是已知的在致癌过程中起重要作用：MIF是促炎细胞因子，他的表达与一些血管生成趋化因子的水平相关，在肺癌组织中有更高的血管密度；14-3-3η蛋白调控许多癌症生物学重要的细胞过程^[8]^。

在不考虑NSCLC亚型的情况下，发现硒结合蛋白1（SELENBP1）、碳酸酐酶（carbonic anhydrase, CA）和肌动蛋白结合蛋白（TAGLN）在癌组织中低表达^[9]^。而硫氧还蛋白（thioredoxin, TXN）、人S100钙结合蛋白A6（S100 calcium binding protein A6, S100A6）、胸腺生成素（thymopoietin, TMPO）、核糖体蛋白L39和S30、过氧化物酶（peroxidase, PRDX）1和3（PRDX1, PRDX3）、烯醇化酶（enolase-1, ENO1）和组蛋白H2A.2在癌组织中过表达^[10]^。

其中，SELENBP1是硒蛋白家族的一员，结合介导胞内的硒运输。SELENBP1的下调在癌症的发生和发展的调控中起主要作用，研究^[11]^表明硒在日常饮食中的缺乏与包括肺癌的上皮癌风险增加相关。

TAGLN是结合肌动蛋白的几种蛋白之一，最近研究^[12]^表明，TAGLN基因作为肿瘤抑制基因，被发现在前列腺癌，乳腺癌和结肠癌中低表达。Rho等^[13]^研究表明TAGLN作为浸润性癌附近的活性基质重塑的标志物，他参与肿瘤-基质相互作用，可能对早期诊断，指导治疗以及监测对治疗反应是有用的。

PRDX是普遍存在的抗氧化酶家族，同时控制着细胞因子诱导的过氧化水平，调控着哺乳动物细胞中的信号传导。PRDX在几种肺部疾病中是高表达的，包括肺癌、间皮瘤和结节病，但是这些变化的机制尚不清楚^[14]^。Park等^[15]^研究表明，在*KRAS*基因驱使的肺腺癌中，通过抑制ROS/ERK/cyclin D1通路的激活，PRDX1作为转录因子NF-E2相关因子2（NF-E2-related factor 2, Nrf2）诱导的肿瘤抑制剂。Tan等^[16]^描述了PRDX2蛋白参与了细胞抗氧化防御，在肺鳞癌中是下调的。

钙网蛋白（S100A8/A9, MRP8/14），是钙结合蛋白S100A8和S100A9的异源二聚体，在多种人类癌症中都可以检测到，高表达于癌细胞和肿瘤浸润免疫细胞。Ohri等^[17]^研究表明在MRP8/14（S100A8/A9）在手术切除的NSCLC标本的非巨噬细胞（non-macrophages, NM）中的过表达。与生存期差的患者相比，NM-MRP8/14在生存期较好的患者中高表达，NM-MRP8/14蛋白可能在肿瘤免疫学和癌症进展中起重要作用^[17]^，有研究^[18]^显示S100A9过表达与NSCLC患者的预后不良有影响。同时S100A8和S100A9在肿瘤样品中低表达。对同一种生物标志物，采用不同样本检测，其表达水平不同，或者对预后的预测存在差异，可能是研究方法及技术手段的不同造成的，也可能是由于S100A8和S100A9单独存在与以异源二聚体的形式存在的确不同的作用或功能。但具体的原因还有待深入的研究。

### 2.2 NSCLC患者体液样品中检测到的诊断性生物标志物

研究^[19]^报道，与对照组相比，血清结合珠蛋白（haptoglobin, HP）水平升高。Park等^[20]^强调了HP α亚基特异的诊断作用，并证明了HP α链在诊断NSCLC时是一个比其他亚基更有前景的生物标志物。

通过蛋白质组学研究还在NSCLC患者中发现了高水平的血清淀粉样蛋白α（serum amyloid A, SAA）^[21]^。SAA属于载脂蛋白家族，在组织损伤或炎症的状态时可能会高达1, 000倍。他也可作为对许多癌症类型的一种非特异性生物标志物^[22]^。Sung等^[23]^强调因为癌症细胞和免疫细胞之间的相互作用，SAA水平的增加与肺癌细胞周围环境中的高水平的细胞因子（白介素1、白介素6）和肿瘤坏死因子-α（tumor necrosis factor-α, TNF-α）是相关联的。最近，SAA1和SAA2被提出可作为肺癌的特异性诊断标志物，因为与健康的志愿者和其他类型的癌症或呼吸道疾病的患者相比，通过液相-串联质谱（liquid chromatography-tandem mass spectrometry, LC MS/MS）、酶联免疫法（enzyme-linked immunosorbent assay, ELISA）和免疫组织化学分析都可以证明SAA1和SAA2在肺癌患者血液和癌症组织样中表达水平更高^[23]^。SAA1和SAA2似乎通过对细胞外基质（extracellular matrix, EMC）的作用和诱导基质金属蛋白酶9（matrix metalloproteinase 9, MMP-9）的表达也参与了肺癌的转移^[23]^。因此，SAA1和SAA2也可以代表新的潜在的抑制肺癌转移的治疗靶点。

Li等^[24]^研究表明，与健康受试者相比，富含亮氨酸的α-2-糖蛋白（LRG1）在癌症患者的尿液样本中高表达。在内皮细胞中，通过启动转化生长因子β（transforming growth factor-β, TGF-β）的信号，LRG1被证明参与促进血管生成。LRG1结合辅助受体内皮因子，并通过ALK1-Smad1/5/8通路促进信号传导。在NSCLC患者的尿液外泌体中高表达的LRG1可能来源于肿瘤组织，LRG1可能是NSCLC的候选生物标志物^[24]^。

对NSCLC及肺部良性疾病患者的血清及胸腔积液研究^[25]^表明，色素上皮衍生因子（pigment epithelium derived factor, PEDF）、凝溶胶蛋白和金属蛋白酶组织抑制因子2（tissue inhibitor of metalloproteinase-2, TIMP2）分别过表达。TIMP2蛋白质通过抑制血管生成因子所促使得组织增殖，可能在维持组织稳态中起关键作用。TIMP2蛋白也与肺实质的破坏相关^[26]^。PEDF是丝氨酸蛋白酶抑制剂超家族的成员。他是一个多功能的分泌蛋白，具有抗血管生成、抗肿瘤生成和神经营养作用。Zhang等^[27]^报道与正常的样本相比，无论在蛋白质水平还是mRNA水平，在NSCLC患者样本中PEDF都是减少的。研究^[27]^指出PEDF的减少与肿瘤微血管密度的增加相关，并与TNM分期、肿瘤大小和总生存率显著相关。

### 2.3 NSCLC组织学类型的诊断性生物标志物

组织学类型是NSCLC最重要的临床特征之一。每个组织学亚型都有其具体的临床特征。在建立一种治疗策略时肿瘤的组织学类型是很重要的，但NSCLC常常显示组织学异质性^[2]^。与NSCLC的组织学相关的分子谱目前还不清楚。对目前已知的部分诊断性的蛋白质组学生物标志物可以根据肺癌的组织学类型进行划分。使用不同的蛋白质组学技术就可以鉴定腺癌的几个诊断标志物。

Seike等^[28]^使用二维差异凝胶电泳技术发现与肿瘤组织学类型高度相关联的几种蛋白。这项研究发现脂肪酸结合蛋白5（fatty acid binding protein 5, FABP5）在区分肺腺癌和肺鳞癌时是有用的。

Kikuchi等^[29]^使用蛋白质组学技术研究了NSCLC两个主要组织学亚型（肺腺癌和肺鳞癌）及正常肺组织的蛋白谱，其中有6个蛋白是特异于肺腺癌（嗜铬粒蛋白B、降钙素相关多肽Ralpha、IPI00911047、神经生长因子、前蛋白转化酶和枯草杆菌蛋白酶），2个蛋白特异于肺鳞癌（视锥蛋白样蛋白1和基质金属蛋白酶10）。

使用二维凝胶电泳及质谱技术分析了肺腺癌组织和癌旁组织的差异表达蛋白，并通过免疫组化验证证明了膜联蛋白1（annexin A1, ANXA1）、锰超氧化物歧化酶2（superoxide dismutase 2, SOD2）及14-3-3 σ在肺腺癌组织和癌旁组织的差异表达^[30]^，丝切蛋白-1（cofilin-1, CFL1）和丙酮酸激酶M2（pyruvate kinase M2, PKM2）在癌组织中显著上调^[31]^。研究表明PKM2和CFL1可作为潜在的诊断和预后标志物以及肺腺癌的治疗靶点。

Li等^[32]^使用膜蛋白质组学研究了肺鳞癌组织和无转移的肺鳞癌肿瘤附近的正常支气管上皮组织，其中，热休克蛋白60（heat shock protein 60, HSP60）在调节细胞分化和增殖中起重要作用，膜联蛋白A5（annexin A5, ANXA5）、膜联蛋白A6（annexin A6, ANXA6）和人γ1肌动蛋白（actin gamma 1, ACTG1）在癌组织中高表达，组织蛋白酶D前蛋白（cathepsin D, CTSD）和铁蛋白重链（ferritin heavy chain 1, FTH1）低表达。这些蛋白质组学研究中所确定的膜联蛋白在细胞信号转导、胞吐作用、炎症、细胞生长和分化过程中发挥着潜在的作用。

### 2.4 SCLC诊断性生物标志物

关于SCLC样本的一些研究鉴定了一些肺癌类型的诊断性生物标记物，对侵袭性的恶性肿瘤的检测可能是有用的。使用不同的蛋白质组技术处理肿瘤组织样品，鉴定出几种蛋白质可以作为SCLC潜在的生物标志物：coactosin样蛋白-1（coactosin-like protein 1, COTL-1）、ACTG1、α-微管蛋白、层粘连蛋白B（laminin B1, LAMB1）、泛素缀合酶E2（ubiquitin-conjugating enzyme, UBE2）、碳酸酐酶1（carbonic anhydrase 1, CA1）^[33]^、β-微管蛋白（β-tubulin）、HSP73、HSP90、核纤层蛋白B（lamin B）、增殖细胞核抗原（proliferating cell nuclear antigen, PCNA）^[34]^，这些蛋白在SCLC的组织样品中过表达的，而钙结合蛋白（S100A6）与邻近的正常组织相比是低表达的^[35]^。一些被鉴定的蛋白，如HSPs和CA，在癌症发展中起重要作用。CA在维持pH平衡上起重要作用。细胞外的酸化（外酸化）与许多肿瘤的进展相关，CA抑制剂可抑制肿瘤细胞系的侵袭能力。HSPs在肿瘤细胞中发挥着稳定许多致癌基因和生长促进蛋白的作用^[36]^。

## 3 预后性生物标志物

预后性生物标志物应与患者的生存率相关。当一个蛋白的表达水平与疾病和肿瘤分期和疾病自然史相关时，他们被认为是具有预测预后的价值。预后性标志物对于鉴定患者能不能进行某一治疗时可能是有用的，但不能预测对该治疗的反应^[5]^。通过免疫组织化学、质谱方法分析和反转录PCR ^[37]^证实，肺癌细胞转移表型似乎与S100A11的表达上调相关。S100A11高表达与较高的TNM分期和淋巴结转移相关^[37]^。

通过比较肺鳞癌组织中有和无淋巴结转移的表现寻找预后性标志物，结果显示ANXA2、细胞角蛋白17（cell keratin 17, CK17）和HSP27低表达^[38]^。ANXA2是钙调节的膜结合蛋白。已确定该蛋白在肿瘤组织中的表达与临床分期和肿瘤分化相关。HSP27的表达与组织病理分化、淋巴结转移、临床分期相关。因此有可能把HSP27作为用于基因治疗HSP27阳性的SCC的潜在靶标。阻断HSP27或HSP27信号通路可能会阻止肺鳞癌的侵袭和转移^[38]^。

超过90%的肺癌死亡是由转移引起的，因此参与这一过程的蛋白是最该考虑的预后标志物^[5]^。研究^[39, 40]^证明与肺腺癌潜在的预后相关的是ANXA3。作为钙调节的膜结合蛋白家族的一员，ANXA3在细胞生长和在信号转导途径上起关键作用。他也是与较晚的临床分期、淋巴结及远处转移、总体生存率低和复发率较高相关^[39, 40]^。

细胞骨架重组是调节细胞迁移和转移的核心过程，在肿瘤发生和发展过程中促进细胞结构重组。很多研究都集中在建立一个有效机制以阻止该过程，以消除转移的发生。参与细胞骨架重排的许多蛋白质已被提出作为癌症中潜在的预后标志物^[5]^。细胞角蛋白是在上皮组织细胞骨架中发现的中间丝结构蛋白。他们被检测时以发生部分降解，以单一蛋白质片段或复合物存在，没有完整的分子结构。在NSCLC的所有组织学类型中都检测到了角蛋白升高。细胞角蛋白19片段（cytokerantin-19-fragment, CYFRA21-1）被证明是在预后和监测肺癌患者是有用的。Park等^[41]^研究证明术前血清中的CYFRA21-1在更高的分期、更大的瘤体及分化更差的肺腺癌患者中水平更高，且这些患者的预后更差。还有研究^[42]^也表明，CK17是NSCLC的一个强力预后因子。

腺癌组织样品的蛋白质组学研究中发现CFL1在某些侵袭性肿瘤中是增加的；PKM2是肿瘤发生过程中一个关键的糖酵解酶，与腺癌患者不良的预后相关^[31]^。

Ⅰ期NSCLC的不良预后也与波形蛋白（vimentin）和非肌球蛋白阳性表达相关。这两种蛋白质与细胞骨架和细胞运动性相关，在过表达时，通过调节细胞运动性提高转移过程。蛋白质组学分析鉴定的这两种分子可能反映细胞转移的功能和恶性肿瘤复发的机制^[43]^。

醛缩酶A（aldolase A, ALDOA）是糖酵解的关键酶，负责催化可逆果糖-1, 6-双磷酸转化为甘油醛-3-磷酸和磷酸二羟丙酮。ALDOA调节各种细胞功能，如肌肉维持、细胞形状和流动等过程。Du等^[44]^报道了ALDOA在肺鳞癌中高度表达，其表达水平与转移、分期、分化程度、预后不良有关，ALDOA降低了细胞运动和肿瘤发生能力。这些数据表明，ALDOA可能是肺鳞癌转移和药物开发的治疗靶点的潜在标记物。

## 4 预测性生物标志物

一个好的预测性生物标记物可以预测某一个特定疗法对特定疾病治疗的效果。建立可变性临床治疗效果，为临床医生有效地选择谁将会受益于特定治疗非常重要，避免不必要的或有害的程序，为个体化治疗提供依据。肺癌蛋白质组学研究相关的预测性标记物目前集中在NSCLC患者对表皮生长因子受体（epidermal growth factor receptor, EGFR）抑制剂的反应。常用的是吉非替尼和厄洛替尼等。通过肺腺癌患者对吉非替尼治疗的不同反应的分析指出，心脏型脂肪酸结合蛋白（heart type fatty acid binding protein, H-FABP）的不同水平与完全和部分缓解相关，H-FABP蛋白参与细胞内脂质的运输、储存和代谢，也可能负责细胞生长和增殖的调节^[45]^。通过基质辅助激光解吸附质谱技术（matrix assisted laser desorption and mass spectrometry, MALDI-MS）对吉非替尼和厄洛替尼治疗的NSCLC患者的血清研究，暴露8个峰信息能够预测治疗的益处^[46]^。基于这些结果创建了一个商业产品Veristrat®（Biodesix, Broom field, CO, USA），目前正处于Ⅲ期临床试验验证阶段，并被用于区分联合厄洛替尼、吉非替尼、贝伐珠单抗或西妥昔单抗治疗的NSCLC患者预后等多项研究^[47, 48]^。SAA1除了可作为一个潜在的诊断性标志物之外^[23]^，最近的研究^[49]^表明SAA1可作为一个预测性生物标记物。

## 5 结论和未来前景

生物标志物在肺癌研究中具有重要的意义。目前绝大多数肺癌仍是在晚期才被确诊，失去了最佳治疗时机，5年生存率非常低^[1]^。随着蛋白质组学技术的发展，开始了对肺癌生物标志物的广泛探索，许多蛋白质分子已被证明了可作为潜在的肺癌生物标志物。但由于敏感性、特异性不高以及缺乏分析性试剂，特别是用于验证质谱数据的抗体或临床试验，使得生物标志物在临床上大规模应用变的举步维艰。同时，临床实验室由于购买新仪器的成本、在大队列人群中获得生物标志物临床实用性的信息、培训专业人员学习新技术以及分析复杂的实验结果等，整合新的蛋白质组学技术依旧很缓慢，目前还没有实现全面的蛋白质组学分析。

对于临床蛋白质组学的研究，主要目的是改善诊断程序、检测预后、预测疗效等，包括对肿瘤细胞生物学功能的精确评估和理解癌症的分子发病机理，以开发新的治疗策略和靶点。虽然目前对肺癌的生物标志物研究众多，但真正能应用临床上还有很长的一段路要走。被定义为一种特异性的生物标志物，可以作为客观测量和评估正常的生理过程、致病过程或对指定的治疗干预的药理学反应的一个指标^[5]^。任何生物标志物应用于临床之前，必须考虑以下几点：①需要具有严格统计标准的大量临床验证，以降低假阳性结果的发生；②单一的生物标记物在预测或监测肺癌时往往也存在假阳性，因此将多个生物标志物的相结合作为临床诊断的标准可能会提高疾病诊断的准确性^[50]^；③研究中获得的生物标记物的重复性也是很重要的，这需要不同的生物样本来验证，不同实验室来证明。

## References

[1] Siegel R, Ma J, Zou Z (2014). Cancer statistics, 2014. CA Cancer J Clin.

[2] Chen Z, Fillmore CM, Hammerman PS (2014). Non-small-cell lung cancers: a heterogeneous set of diseases. Nat Rev Cancer.

[3] Agulló-Ortuño MT, López-Ríos F, Paz-Ares L (2010). Lung cancer genomic signatures. J Thorac Oncol.

[4] Herbst RS, Heymach JV, Lippman SM (2008). Lung cancer. N Engl J Med.

[5] Kisluk J, Ciborowski M, Niemira M (2014). Proteomics biomarkers for non-small cell lung cancer. J Pharm Biomed Anal.

[6] Campa MJ, Wang MZ, Howard B (2003). Protein expression profiling identifies macrophage migration inhibitory factor and cyclophilin a as potential molecular targets in non-small cell lung cancer. Cancer Res.

[7] Xiao T, Ying W, Li L (2005). An approach to studying lung cancer-related proteins in human blood. Mol Cell Proteomics.

[8] Rahman SM, Gonzalez AL, Li M (2011). Lung cancer diagnosis from proteomic analysis of preinvasive lesions. Cancer Res.

[9] Li LS, Kim H, Rhee H (2004). Proteomic analysis distinguishes basaloid carcinoma as a distinct subtype of non-small cell lung carcinoma. Proteomics.

[10] Yanagisawa K, Tomida S, Shimada Y (2007). A 25-signal proteomic signature and outcome for patients with resected non-small-cell lung cancer. J Natl Cancer Inst.

[11] Zeng GQ, Yi H, Zhang PF (2013). The function and significance of SELENBP1 down regulation in human bronchial epithelial carcinogenic process. PLoS One.

[12] Assinder SJ, Stanton JA, Prasad PD (2009). Transgelin: an actin-binding protein and tumour suppressor. Int J Biochem Cell Biol.

[13] Rho J, Roehrl MH, Wang JY (2009). Tissue proteomics reveals differential and compartment-specific expression of the homologs transgelin and transgelin-2 in lung adenocarcinoma and its stroma. J Proteome Res.

[14] Schremmer B, Manevich Y, Feinstein SI (2007). Peroxiredoxins in the lung with emphasis on peroxiredoxin Ⅵ. Subcell Biochem.

[15] Park YH, Kim SU, Lee BK (2013). Prx I suppresses K-ras-driven lung tumorigenesis by opposing redox-sensitive ERK/cyclin D1 pathway. Antioxid Redox Signal.

[16] Tan F, Jiang Y, Sun N (2012). Identification of isocitrate dehydrogenase 1 as a potential diagnostic and prognostic biomarker for non-small cell lung cancer by proteomic analysis. Mol Cell Proteomics.

[17] Ohri CM, Shikotra A, Green RH (2011). The tissue microlocalisation and cellular expression of CD163, VEGF, HLA-DR, iNOS, and MRP 8/14 is correlated to clinical outcome in NSCLC. PLoS One.

[18] Kawai H, Minamiya Y, Takahashi N (2011). Prognostic impact of S100A9 overexpression in non-small cell lung cancer. Tumor Biol.

[19] Ulivi P, Mercatali L, Casoni GL (2013). Multiple marker detection in peripheral blood for NSCLC diagnosis. PLoS One.

[20] Park J, Yang JS, Jung G (2013). Subunit-specific mass spectrometry method identifies haptoglobin subunit alpha as a diagnostic marker in non-small cell lung cancer. J Proteomics.

[21] Kanoh Y, Abe T, Masuda N (2013). Progression of non-small cell lung cancer: diagnostic and prognostic utility of matrix metalloproteinase-2, C-reactive protein and serum amyloid A. Oncol Rep.

[22] Li J, Xie Z, Shi L (2012). Purification, identification and profiling of serum amyloid A proteins from sera of advanced-stage cancer patients. J Chromatogr B Analyt Technol Biomed Life Sci.

[23] Sung HJ, Ahn JM, Yoon YH (2011). Identification and validation of SAA as a potential lung cancer biomarker and its involvement in metastatic pathogenesis of lung cancer. J Proteome Res.

[24] Li Y, Zhang Y, Qiu F (2011). Proteomic identification of exosomal LRG1: apotential urinary biomarker for detecting NSCLC. Electrophoresis.

[25] Rodriguez-Pineiro AM, Blanco-Prieto S, Sanchez-Otero N (2010). On the identification of biomarkers for non-small cell lung cancer in serum and pleural effusion. J Proteomics.

[26] Garbacki N, Di Valentin E, Piette J (2009). Matrix metalloproteinase 12 silencing: a therapeutic approach to treat pathological lung tissue remodeling?. Pulm Pharmacol Ther.

[27] Zhang L, Chen J, Ke Y (2006). Expression of pigment epithelial derived factor is reduced in non-small cell lung cancer and is linked to clinical outcome. Int J Mol Med.

[28] Seike M, Kondo T, Fujii K (2005). Proteomic signatures for histological types of lung cancer. Proteomics.

[29] Kikuchi T, Hassanein M, Amann JM (2012). In-depth proteomic analysis of non-small cell lung cancer to discover molecular targets and candidate biomarkers. Mol Cell Proteomics.

[30] Xiao G, Lu Q, Li C (2010). Comparative proteome analysis of human adenocarcinoma. Med Oncol.

[31] Peng X, Gong F, Zhao Y (2011). Comparative proteomic approach identifies PKM2 and cofilin-1 as potential diagnostic, prognostic and therapeutic targets for pulmonary adenocarcinoma. PLoS One.

[32] Li B, Chang J, Chu Y (2012). Membrane proteomic analysis comparing squamous cell lung cancer tissue and tumor-adjacent normal tissue. Cancer Lett.

[33] Jeong HC, Kim GI, Cho SH (2010). Proteomic analysis of human small cell lung cancer tissues: up-regulation of coactosin-like protein-1. J Proteome Res.

[34] Okuzawa K, Franzén B, Lindholm J (1994). Characterization of gene expression in clinical lung cancer materials by two-dimensional polyacrylamide gel electrophoresis. Electrophoresis.

[35] Lee HS, Park JW, Chertov O (2012). Matrix-assisted laser desorption/ionization mass spectrometry reveals decreased calcylcin expression in small cell lung cancer. Pathol Int.

[36] Trepel J, Mollapour M, Giaccone G (2010). Targeting the dynamic HSP90 complex in cancer. Nat Rev Cancer.

[37] Tian T, Hao J, Xu A (2007). Determination of metastasis-associated proteins in non-small cell lung cancer by comparative proteomic analysis. Cancer Sci.

[38] Yao H, Zhang Z, Xiao Z (2009). Identification of metastasis associated proteins in human lung squamous carcinoma using two-dimensional difference gel electrophoresis and laser capture microdissection. Lung Cancer.

[39] Liu YF, Chen YH, Li MY (2012). Quantitative proteomic analysis identifying three annexins as lymph node metastasis-related proteins in lung adenocarcinoma. Med Oncol.

[40] Liu YF, Xiao ZQ, Li MX (2009). Quantitative proteome analysis reveals annexin A3 as a novel biomarker in lung adenocarcinoma. J Pathol.

[41] Park SY, Lee JG, Kim J (2013). Preoperative serum CYFRA 21-1 level as a prognostic factor in surgically treated adenocarcinoma of lung. Lung Cancer.

[42] De Petris L, Brandén E, Herrmann R (2011). Diagnostic and prognostic role of plasma levels of two forms of cytokeratin 18 in patients with non-small-cell lung cancer. Eur J Cancer.

[43] Xu B J, Gonzalez A L, Kikuchi T (2008). MALDI‐MS derived prognostic protein markers for resected non‐small cell lung cancer. Proteomics Clin Appl.

[44] Du S, Guan Z, Hao L (2014). Fructose-bisphosphate aldolase a is a potential metastasis-associated marker of lung squamous cell carcinoma and promotes lung cell tumorigenesis and migration. PLoS One.

[45] Okano T, Kondo T, Fujii K (2007). Proteomic signature corresponding to the response to gefitinib (Iressa, ZD1839), an epidermal growth factor receptor tyrosine kinase inhibitor in lung adenocarcinoma. Clin Cancer Res.

[46] Taguchi F, Solomon B, Gregorc V (2007). Mass spectrometry to classify non-small-cell lung cancer patients for clinical outcome after treatment with epidermal growth factor receptor tyrosine kinase inhibitors: a multicohort cross-institutional study. J Natl Cancer Inst.

[47] Molina-Pinelo S, Pastor MD, Paz-Ares L (2014). VeriStrat: a prognostic and/or predictive biomarker for advanced lung cancer patients?. Expert Rev Respir Med.

[48] Gautschi O, Dingemans AM, Crowe S (2013). VeriStrat(R) has a prognostic value for patients with advanced non-small cell lung cancer treated with erlotinib and bevacizumab in the first line: Pooled analysis of SAKK19/05 and NTR528. Lung cancer.

[49] Milan E, Lazzari C, Anand S (2012). SAA1 is over-expressed in plasma of non- small cell lung cancer patients with poor outcome after treatment with epidermal growth factor receptor tyrosine-kinase inhibitors. J Proteomics.

[50] Sun T, Simon I, Moreno V (2013). A combined oncogenic pathway signature of BRAF, KRAS and PI3KCA mutation improves colorectal cancer classification and cetuximab treatment prediction. Gut.

